# Tuberculosis infection of the breast mistaken for granulomatous mastitis: a case report

**DOI:** 10.1186/1757-1626-1-273

**Published:** 2008-10-25

**Authors:** KB Sriram, D Moffatt, R Stapledon

**Affiliations:** 1 Department of Thoracic Medicine, Royal Adelaide Hospital, Adelaide, South Australia 5000, Australia

## Abstract

**Background:**

Tuberculosis of the breast is an uncommon disease with non-specific clinical, radiological and histological findings. Misdiagnosis is common as biopsy specimens are pauci-bacillary and investigations such as microscopy and culture are frequently negative.

**Case presentation:**

We report a case of a breast abscess in a 34-year old Bangladeshi woman attributed to tuberculosis infection. Equivocal histology, negative Ziehl-Neelsen stain and culture for acid-fast bacilli resulted in the abscess initially being diagnosed as granulomatous mastitis and treated accordingly. However failure to respond to therapy raised suspicion of culture negative breast tuberculosis. Treatment with standard antituberculosis drugs was associated with complete resolution of the breast abscess.

**Conclusion:**

This case highlights the difficulty in differentiating culture negative tuberculosis from granulomatous mastitis and the importance of a high index of clinical suspicion.

## Introduction

Breast tuberculosis (TB) is a rare disease, with an incidence of less than 0.1% of all breast lesions in Western countries and 4% of all breast lesions in TB endemic countries [1,2]. It typically affects young lactating multiparous women and can present either as an abscess or as a unilateral, painless breast mass [1,2]. Breast TB is paucibacillary and consequently tests such as microscopy, culture and nucleic acid amplification tests such as polymerase chain reaction techniques do not have the same diagnostic utility as they do in pulmonary tuberculosis [3]. Thus, it is not uncommon for breast TB to be misdiagnosed either as non-specific abscess or carcinoma [4,5]. We report a patient with a presumed TB breast abscess that was initially diagnosed and treated as granulomatous mastitis abscess.

## Case report

**Figure 1:**
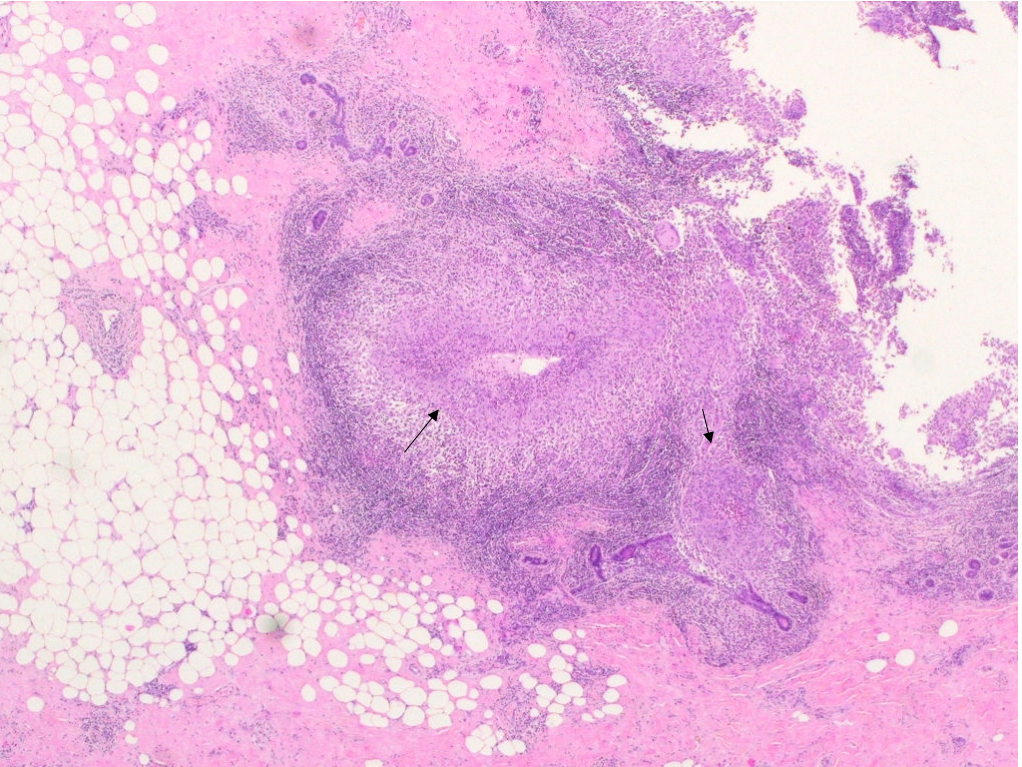
**Low power field of an excision biopsy of the breast mass showing a mixed inflammatory cell infiltrate (block arrow) with suppurative granulomas (thin arrow).** (haematoxylin and eosin stain; original magnification × 40).

**Figure 2:**
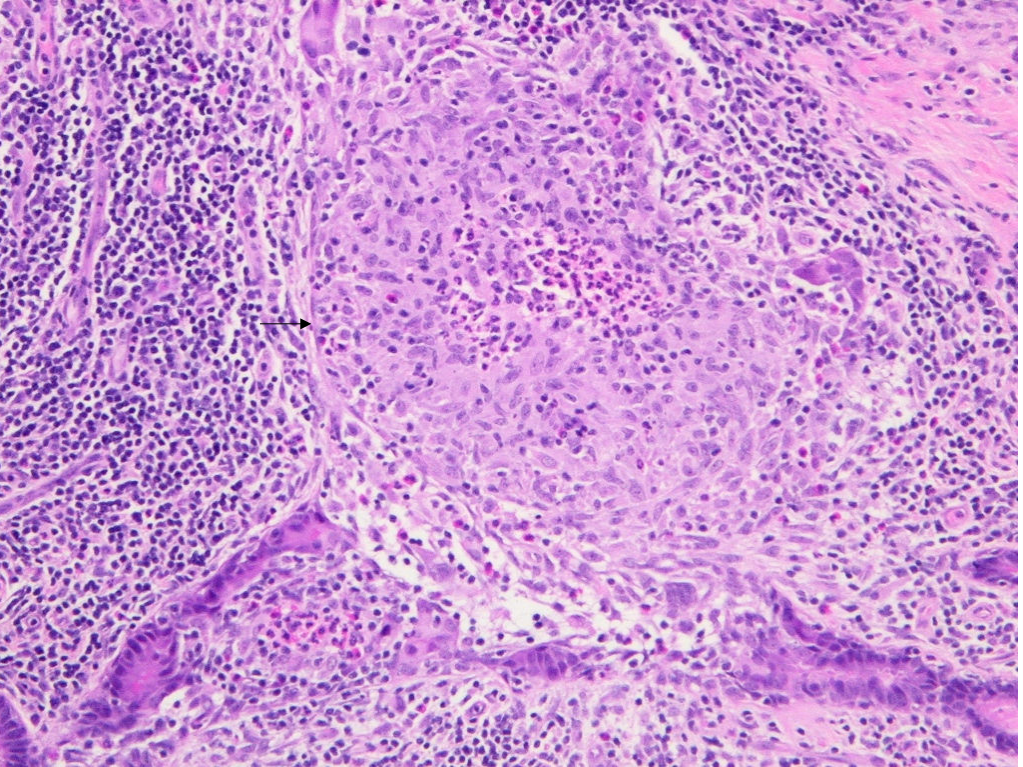
**High power field showing suppurative granuloma including giant cells (arrow).** The granulomatous inflammation is centred on ducts and lobules (haematoxylin and eosin stain; original magnification × 200).

A 34-year old HIV negative woman presented for evaluation of an abscess in her right breast which developed one month prior to presentation and was associated with pain and tenderness. She denied fever, night sweats, weight loss or respiratory symptoms. There was no family history of breast cancer and no personal history of diabetes, immunosuppression, previous treatment for tuberculosis or recent exposure to a person with tuberculosis. Right axillary lump removed in 2000 the nature of which was unclear. She had migrated to Australia from Bangladesh 6 years ago. She had one five-year-old child and had ceased breast-feeding three years prior. She was not pregnant at the time of presentation and denied recent use of hormonal contraception. On examination, she had a 12 × 9 cm firm mass in the upper quadrant of her right breast and no associated palpable adenopathy. There was some nipple inversion but no discharge. Complete blood picture showed a total white cell count of 15 × 10^9^/L(normal range 4–11 × 10^9^/L) and C-reactive protein of 72 mg/L (normal < 10 mg/L). Ultrasonography of the right breast lump showed a diffuse hypoechoic abnormality in the upper central aspect. Mammography showed increased density and coarsened trabeculation but no microcalcification or suspicious focal abnormalities. An excision biopsy of the breast mass was performed which showed granulomatous inflammation in a mixed inflammatory cell background consisting of lymphocytes, plasma cells and polymorphs. The granulomas were within the ducts and caseous necrosis was not identified (Figure 1 and 2). There was no evidence of atypical epithelial hyperplasia or malignancy. Gram stain, Z-N stain, PAS-D stain were negative but bacteriological cultures grew *Corynebacterium kroppenstedtii*. A chest x-ray did not suggest current or previous TB disease.

The patient was treated with doxycycline for suspected granulomatous mastitis abscess. During six weeks of antibiotic therapy there was sinus formation and discharge of foul smelling purulent material. Based on patient profile, histological findings and lack of clinical response to antibiotic therapy, *M. tuberculosis *was considered the most likely causative pathogen for the breast abscess. Standard 6-month anti TB therapy (isoniazid, rifampicin, pyrazinamide and ethambutol) was commenced with good clinical response. Two months after completion of anti TB therapy, no breast mass was palpable, total white cell count was 8.71 × 10^9^/L and C-reactive protein was 4 mg/L. Mammogram and ultrasound confirmed resolution of the mass lesion with residual scar tissue only. Two years after completion of therapy she remains asymptomatic with no recurrence of abscess.

## Discussion

The differential diagnosis of granulomatous inflammation in the breast includes other infections (culture negative and stains for organisms performed on the sections – PAS-D and gram as well as ZN were all negative), sarcoidosis (suppuration not typical and no supportive clinical features), granulomatous reaction to tumour (no evidence of malignancy clinically, radiologically or pathologically), and foreign body reaction (no histological foreign body identified). Mammary duct ectasia could be considered in the differential but epithelioid granulomas are not a usual feature of this disease: the macrophages tend to be foamy in appearance and multinucleate giant cells tend to be associated with cholesterol crystals. Wegener's can be excluded in the absence of a necrotising vasculitis. The granulomas were seen to be centred around ducts rather than lobules, but in the larger more destructive granulomas it was difficult to work out any spatial relationship with any structures. TB is associated with ducts more than lobules, lending more support to TB rather than granulomatous mastitis in this particular case.

Granulomatous mastitis is uncommon, can develop a few years after the woman's last child birth and can present either as a palpable tender mass or as an abscess [6-8]. While pathogenesis is unclear associations have been reported with breast feeding, oral contraceptive use and infection with *Corynebacterium *spp. [6]. The diagnosis is made histologically by identifying lobular chronic non-caseating granulomatous inflammation with giant cells, leucocytic infiltrations, foamy macrophages and abscesses. However these findings also overlap with breast TB and thus to diagnose granulomatous mastitis *M. tuberculosis *has to be definitively excluded. While routine treatment consists of steroids and complete surgical excision, antibiotic therapy is recommended if granulomatous mastitis presents as an abscess. Despite appropriate therapy, granulomatous mastitis can recur in up to 50% usually within 6 weeks to 11 months after stopping treatment [6].

Breast TB commonly presents as a lump in the central or upper outer quadrant of the breast. Studies have shown that a high proportion of breast TB patients do not present with pulmonary or systemic symptoms[1,2]. Breast TB is classified as nodular, disseminated and abscess varieties. Tubercular breast abscess, though less common than the nodular form, is a common mode of presentation in young women. Breast TB can either be primary when the breast lesion is the only manifestation of TB or secondary where there is a demonstrable focus of TB elsewhere [1,2]. Primary breast TB is considered rare and it is assumed that most cases are secondary even if no primary focus can be found [2]. In our patient a primary focus of infection was not found and histologically there was lack of suppuration and caseous necrosis suggesting primary infection. However we exercise caution in reaching this conclusion because she migrated from a TB endemic country and we cannot exclude lack *M. tuberculosis *as the aetiology of the axillary lump that was excised six years prior to presentation.

The mammogram has limited utility in diagnosis of breast TB as the findings are indistinguishable from carcinoma. Additionally breast TB typically affects young women, whose dense breasts are difficult to analyse mammographically [2]. Ultrasonography helps to better define the lesion and improve the success rate of fine needle aspiration cytology (FNAC), rather than provide definitive diagnosis.

FNAC from the breast lesion can diagnose breast TB in as many as three quarters of cases when both epitheloid cell granulomas and necrosis are present [2]. However failure to demonstrate necrosis on FNAC does not exclude TB as often a spectrum of histological abnormalities can be found in breast TB specimens [2]. Mycobacterial culture, the gold standard for the diagnosis of TB, is often negative due to the paucibacillary nature of breast TB [2]. Nucleic acid amplification tests (NAAT) such as polymerase chain reaction (PCR) are rapid and specific but suffer from low sensitivity especially in AFB smear negative cases. Sensitivity as low as 50% have been reported in some series [9]. Further complicating the issue is the presence of polymerase enzyme inhibitors in approximately 20% of extrapulmonary specimens [9]. If formalin fixed tissue is the only available material sensitivity of NAAT is compromised further. Thus a negative NAAT result does not exclude TB disease with certainty. The Tuberculin skin test, interferon gamma release assays and serology are of limited diagnostic value given that adults from TB endemic areas are expected to have high rates of positivity for these tests [10,11].

Treatment of breast TB with standard antituberculosis therapy for 6 months usually results in good clinical response [1,2,4,5,9]. The regimen consists of a two month intensive phase (isoniazid, rifampicin, pyrazinamide and ethambutol) followed by a four month continuation phase (isoniazid and rifampicin). Surgical intervention is only necessary if there is poor response to anti-TB therapy, and is reserved for draining cold abscesses or excision of residual lumps. Simple mastectomy with or without axillary clearance is reserved for cases with extensive disease causing a large painful ulcerated mass involving the entire breast [2].

Our patient had a significant risk for past TB infection as she originated from Bangladesh, where the estimated incidence of TB in 2005 was 227 per 100,000 population [12]. Although TB was considered, the histological findings of granulomas without necrosis and microbiological growth of *Corynebacterium *spp led to a diagnosis of granulomatous mastitis abscess. Failure to respond to appropriate antibiotic therapy prompted review of this diagnosis. A standard six-month course of antituberculosis therapy resulted in a good clinical and radiological response with complete resolution of the breast abscess and no evidence of relapse after two years.

In conclusion breast TB should be suspected when there is poor response to (non TB) antibiotics used for treatment of breast abscess, especially in a young patient originating from a TB endemic country. If there is a high clinical suspicion of TB, then a trial of antituberculosis therapy with regular clinical assessment is warranted.

## Abbreviations

TB: Tuberculosis; Z-N: Ziehl-Neelsen; FNAC: Fine needle aspiration cytology; FNA: Fine needle aspiration; PAS-D: Periodic acid Schiff-diastase.

## Consent

Written informed consent was obtained from the patient for publication of this case report and accompanying images. A copy of the written consent is available for review by the Editor-in-Chief of this journal.

## Competing interests

The authors declare that they have no competing interests.

## Authors' contributions

KBS reviewed the case notes and prepared the manuscript. RS and DM read and approved the final manuscript.
